# Topical Application of Apricot Kernel Extract Improves Dry Eye Symptoms in a Unilateral Exorbital Lacrimal Gland Excision Mouse

**DOI:** 10.3390/nu8110750

**Published:** 2016-11-23

**Authors:** Chan-Sik Kim, Kyuhyung Jo, Ik-Soo Lee, Junghyun Kim

**Affiliations:** Korean Medicine Convergence Research Division, Korea Institute of Oriental Medicine, Daejeon 34054, Korea; chskim@kiom.re.kr (C.-S.K.); jopd7414@kiom.re.kr (K.J.); knifer48@kiom.re.kr (I.-S.L.)

**Keywords:** apricot kernel, dry eye, mucin, tear

## Abstract

The purpose of this study was to investigate the therapeutic effects of topical application of apricot kernel extract (AKE) in a unilateral exorbital lacrimal gland excision mouse model of experimental dry eye. Dry eye was induced by surgical removal of the lacrimal gland. Eye drops containing 0.5 or 1 mg/mL AKE were administered twice a day from day 3 to day 7 after surgery. Tear fluid volume and corneal irregularity scores were determined. In addition, we examined the immunohistochemical expression level of Muc4. The topical administration of AKE dose-dependently improved all clinical dry eye symptoms by promoting the secretion of tear fluid and mucin. Thus, the results of this study indicate that AKE may be an efficacious topical agent for treating dry eye disease.

## 1. Introduction

Dry eye syndrome is a common complaint in the elderly and an uncomfortable condition induced by inadequate or altered tear film stability, which results from the inability of the lacrimal glands to generate a sufficient quantity of tear fluid [1]. Tear film instability leads to visual disturbances, inflammation and injury to the ocular surface [2].

The first treatment option for dry eye is the use of artificial tears. However, artificial tears only partially replace the aqueous tear fluid components, and multiple topical administrations are often needed. Eye drops made from hyaluronate sodium or autologous serum have provided marginal clinical benefit in patients with dry eye syndrome [3,4,5]. Several compounds, such as OPC-127959 and diquafosol sodium, facilitate the production of tear fluid and mucin and have improved patients’ dry eye symptoms [6,7]. Although these topical medications have shown some efficacy in improving ocular surface integrity, the long-term use of these medications sometimes leads to adverse drug reactions, such as ocular hyperemia, eye irritation and corneal calcification [8]. Therefore, it is necessary to identify novel safe agents with enhanced effectiveness for the treatment of dry eye.

The interest in natural products for drug development has been growing because of the possible negative effects of synthetic compounds. *Prunus armeniaca* (apricot) is a Rosaceae plant that grows in China, Japan, Turkey and Korea. The fresh and dried fruit of this plant have been used as a food worldwide. The apricot kernel is the seed of *Prunus armeniaca* and is known to have many pharmacological benefits for managing thirst, cough and fever [9]. In several Asian countries, apricot kernels have been traditionally used as a botanical drug to treat asthma, bronchitis, constipation, emphysema, nausea, leprosy and leukoderma [10]. Although various pharmacological effects of apricot kernel have been reported, its effect on dry eye disease remains unknown. Therefore, in this study, we investigated the therapeutic effect of an extract of apricot kernel on dry eye and identified its possible mechanism of action in a mouse model established by exorbital lacrimal gland excision.

## 2. Materials and Methods

### 2.1. Preparation of Apricot Kernel Extract

A standardized apricot kernel extract (AKE) was obtained from a plant extract bank at the Korea Research Institute of Bioscience & Biotechnology (Daejeon, Korea). Briefly, dried and ground seed kernel of *P. armeniaca* (500 g) was boiled with distilled water at 100 °C for 2 h, and the extract was condensed using freeze-drying (yield: 6.9%). The AKE was standardized using a reference compound, amygdalin (Sigma, St. Louis, MO, USA), by high-performance liquid chromatography (HPLC) according to previously reported protocols [11]. Briefly, the AKE (10 mg) was dissolved in 20% methanol (10 mL) and then the solution was filtered through a 0.2 μm syringe filter (Millipore, Bedford, MA, USA) prior to injection. An amygdalin standard stock solution of 1 mg/mL was prepared in 20% methanol and stored at a temperature below 4 °C. Calibration standard solutions at five levels were prepared by serially diluting the stock solution to concentrations of 12.5, 25, 50, 100, and 200 μg/mL. The solutions were filtered through a 0.2 μm syringe filter (Millipore) prior to injection. Each analysis was repeated three times, and the calibration curves were fitted by linear regression. HPLC analysis was performed with an Agilent 1200 HPLC instrument (Agilent Technologies, Santa Clara, CA, USA) equipped with a binary pump, vacuum degasser, auto-sampler, column compartment, and diode array detector (DAD). The column used was a Luna C18 (250 × 4.6 mm, 5.0 μm, Phenomenex, Torrance, CA, USA). The mobile phase consisted of acetonitrile and water (22:78, v:v). Column temperature was maintained at 30 °C. Analysis was performed at a flow rate of 1.0 mL/min for 20 min and monitored at 214 nm. The injection volume of the sample was 10 µL.

### 2.2. Animals and Experimental Design

Seven-week-old female C57BL/6 mice were purchased from Orient Bio (Seoul, Korea). The mice were deeply anesthetized with pentobarbital sodium (30 mg/kg body weight; Hanlim Pharmaceuticals Co., Ltd., Seoul, Korea). Experimental dry eye was induced by surgical excision of the left exorbital lacrimal gland. Mice in the normal control group (NOR) were maintained without surgical operation. At three days after surgery, the exorbital lacrimal gland excised mice were randomly assigned to three groups: (1) vehicle-treated dry eye mice (DE); (2) 0.5 mg/mL AKE-treated DE mice (AKE-0.5) and (3) 1 mg/mL AKE-treated DE mice (AKE-1). AKE ophthalmic solution at 0.05% and 0.1% were prepared as a sterile isotonic aqueous solution (pH 7.4) in balanced salt solution (BSS, Alcon, Fort Worth, TX, USA). The osmolality of the AKE ophthalmic solution was 290 mOsmol/kg. A total of 20 µL of 0.5 or 1 mg/mL AKE solution was topically applied to each eye of the exorbital lacrimal gland excised mice twice daily for five days. Mice in the NOR and DE groups received 20 µL of balanced salt solution (vehicle). AKE eye drops were administered directly onto the superior corneal surface of each eye. There is no specific sign of side effects in any group after the topical application of AKE, The animal experiments were conducted according to a procedure approved by our Institutional Animal Care and Use Committee (IACUC approval No. 15-059).

### 2.3. Tear Measurement

Tear volume was measured at day 3 and day 7 after surgery. Phenol red-impregnated cotton threads (Zone Quick; FCI Ophthalmics, Pembroke, MA, USA) were held with fine forceps and placed in the lateral canthus for 30 s. The tear volume was measured under a microscope and expressed in terms of the length (in millimetres) of color-changed thread that absorbed the tear fluid.

### 2.4. Corneal Fluorescein Staining

Corneal fluorescein staining was used to evaluate corneal epithelial damage. A dose of 1 µL of 2.5% fluorescein (Sigma, St. Louis, MO, USA) was administered into the lateral conjunctival sac of the mice, and 3 min later, their corneas were examined using a slit-lamp biomicroscope (Kowa, Tokyo, Japan) under cobalt blue light. A corneal fluorescein grading score (0: none, 1: mild, 2: moderate and 3: severe) was assigned to three different corneal areas (upper, middle, and lower areas). A score of nine points indicated severe keratitis.

### 2.5. Corneal Irregularity Analysis

Corneal irregularity was examined in mice from each group as previously described [12]. Briefly, reflected lines of a ring-shape light from the fiber-optic ring illuminator of a stereomicroscope (SZ51; Olympus, Tokyo, Japan) were projected on the corneal surface after anesthesia, and the reflected lines of light were captured with a DP21 digital camera (Olympus). Scores of corneal irregularity were graded according to the number of distorted quadrants in the reflected white ring as follows: 0, no distortion; 1, distortion in 1 quadrant; 2, distortion in 2 quadrants; 3, distortion in 3 quadrants; 4, distortion in all 4 quadrants; 5, severe distortion in which no ring could be recognized.

### 2.6. Immunohistochemistry

To investigate the expression levels of Muc4, immunohistochemical staining was performed according to previously reported protocols [13]. Briefly, the antibody used in this study was mouse anti-Muc4 antibody (Cell Signaling, Danvers, MA, USA). To detect Muc4, the slides were labeled with a LSAB kit (DAKO, Carpinteria, CA, USA) and visualized with a DAB substrate kit (DAKO). For morphometric analyses, the immunoreactive intensity per unit area (mm^2^) was measured using ImageJ software (NIH, Bethesda, MD, USA).

### 2.7. Western Blot Analysis

Corneal and conjunctival tissues were homogenized in RIPA buffer (150 mM NaCl, 1.0% IGEPAL CA-630, 0.5% sodium deoxycholate, 0.1% sodium dodecyl sulfate, 50 mM Tris, pH 8.0, 1× protease inhibitors, and 1 mM phenylmethylsulfonyl fluoride). Homogenates were centrifuged at 14,000× *g* for 20 min, and total protein concentrations were determined with the Bio-Rad Protein Assay kit (Bio-Rad Laboratories, Hercules, CA, USA). Proteins were analyzed using sodium dodecyl sulfate–polyacrylamide gel electrophoresis and transferred to polyvinylidene difluoride membranes (Bio-Rad, Hercules, CA, USA). The membranes were labeled with mouse anti-TNF-α (Santa Cruz Biotechnology, Paso Robles, CA, USA) and mouse anti-β-actin antibodies (Sigma, St. Louis, MO, USA). The immunoreactive bands were detected using chemiluminescence detection reagents (Pierce, Rockford, IL, USA), and the density of the bands of interest was measured using a LAS-3000 machine (Fujifilm, Tokyo, Japan). Anti-β-actin antibody served as a loading control.

### 2.8. Statistical Analysis

Significant differences between groups were analyzed using one-way analysis of variance (ANOVA) and Tukey’s multiple comparison test in the Prism 5.0 software (GraphPad, La Jolla, CA, USA), and *p* < 0.05 was considered statistically significant.

## 3. Results

### 3.1. Standardization of AKE

**Figure 1:**
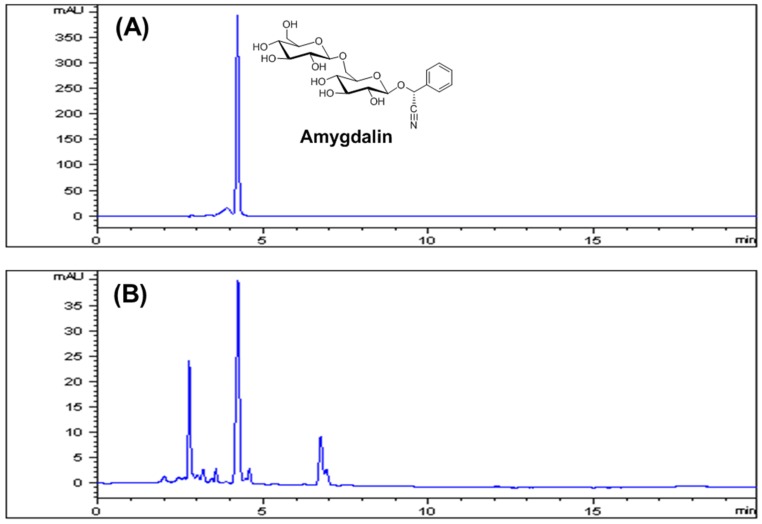
HPLC chromatographs of an amygdalin standard (**A**) and an extract of apricot kernel (**B**) with detection at 214 nm. The peak of amygdalin appeared at a retention time of 4.20 min.

Amygdalin (d-mandelonitrile-2-d-gentiobioside) is a cyanogenic glycoside present in apricot kernel and is known as its major ingredient [14]. To ensure the quality of the AKE, an HPLC-based method was used to quantitatively analyze amygdalin in the AKE. The identification and quantitative determination of amygdalin in the extract were accomplished by comparing the retention time and area with those of standard amygdalin (Figure 1). The linearity of the HPLC method was assessed by injecting five concentrations of standard amygdalin solution. The calibration curve showed good linearity (*R*^2^ = 0.9999) in the range of 12.5–200 μg/mL of the amygdalin standard (Table 1). The content of amygdalin in the AKE was 127.34 ± 0.99 mg/g (Table 2).

### 3.2. AKE Improves Tear Secretion

**Figure 2:**
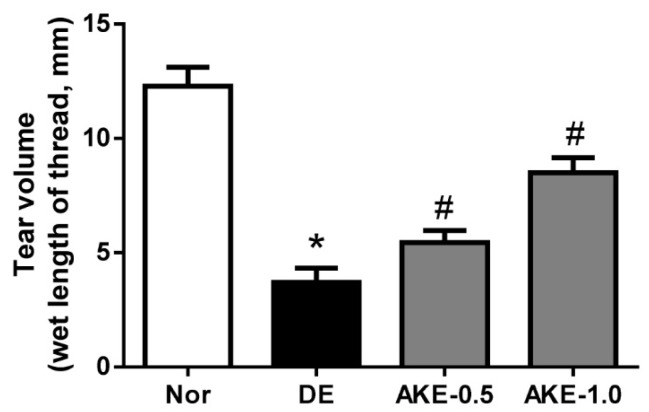
Effects of AKE on aqueous tear secretion. Tear volume was measured using the phenol red thread tear test. Tear volume was expressed in millimeters of thread that became wet by the tear and turned red in color. NOR: normal control mice, DE: vehicle-treated dry eye mice, AKE-0.5: 0.5 mg/mL AKE ophthalmic solution-treated DE mice, and AKE-1.0: 1 mg/mL AKE ophthalmic solution-treated DE mice. The values in the bar graph represent the mean ± standard error (SE), *n* = 10. * *p* < 0.05 vs. normal mice, # *p* < 0.05 vs. vehicle-treated dry eye mice.

To investigate the effect of AKE on tear fluid secretion, we performed the phenol red thread tear test in the exorbital lacrimal gland–excised mice. Three days after the removal of the exorbital lacrimal gland, all of the dry eye mice exhibited significantly reduced tear volume compared with normal control mice (*p* < 0.05). However, topical application of AKE significantly increased aqueous tear volume at five days after application (*p* < 0.05). As shown in Figure 2, the mean tear volumes were 3.69 ± 1.79 mm in the vehicle-treated DE group and 5.44 ± 1.50 mm and 8.50 ± 2.08 mm in the AKE-treated groups. Mice treated with AKE exhibited 20.3% and 55.9% increases in tear volume compared with the tear volume in the vehicle-treated DE mice. These results indicate that AKE helps to increase tear secretion.

### 3.3. AKE Prevents Corneal Epithelial Damage

**Figure 3:**
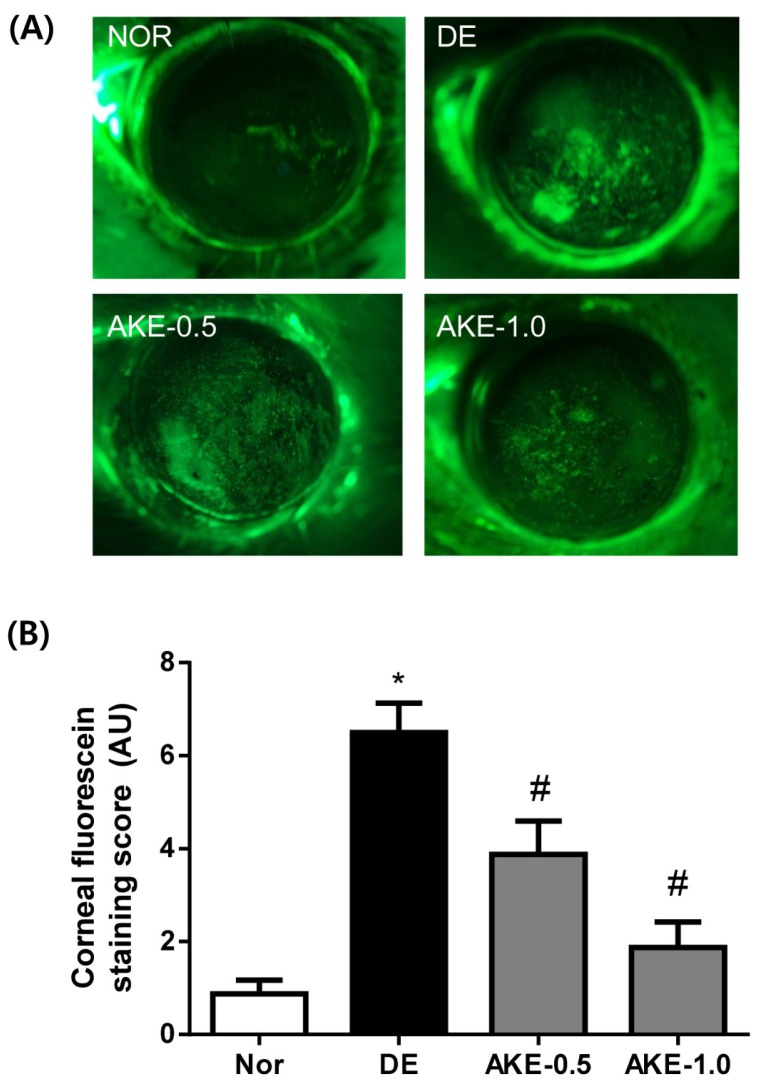
Effects of AKE on corneal epithelial damage. (**A**) Fluorescent slit-lamp photographs of mouse eyes with or without AKE treatment; (**B**) Corneal fluorescein grading score after the topical application of AKE. The values in the bar graph represent the mean ± SE, *n* = 10. * *p* < 0.05 vs. normal mice, # *p* < 0.05 vs. vehicle-treated dry eye mice.

As shown in Figure 3A,B, the corneal fluorescein staining scores significantly increased in the DE group (6.50 ± 1.77) compared with those of the NOR group (0.88 ± 0.83, *p* < 0.05). Five days after the topical application of AKE, the mean corneal fluorescein scores of the two treatment groups were 3.88 ± 2.03 and 1.87 ± 1.55. This effect of AKE was statistically significant compared with the finding in the DE group (*p* < 0.05). These findings suggest that AKE is useful in treating experimental dry eye conditions.

### 3.4. AKE Inhibits Corneal Regularization

**Figure 4:**
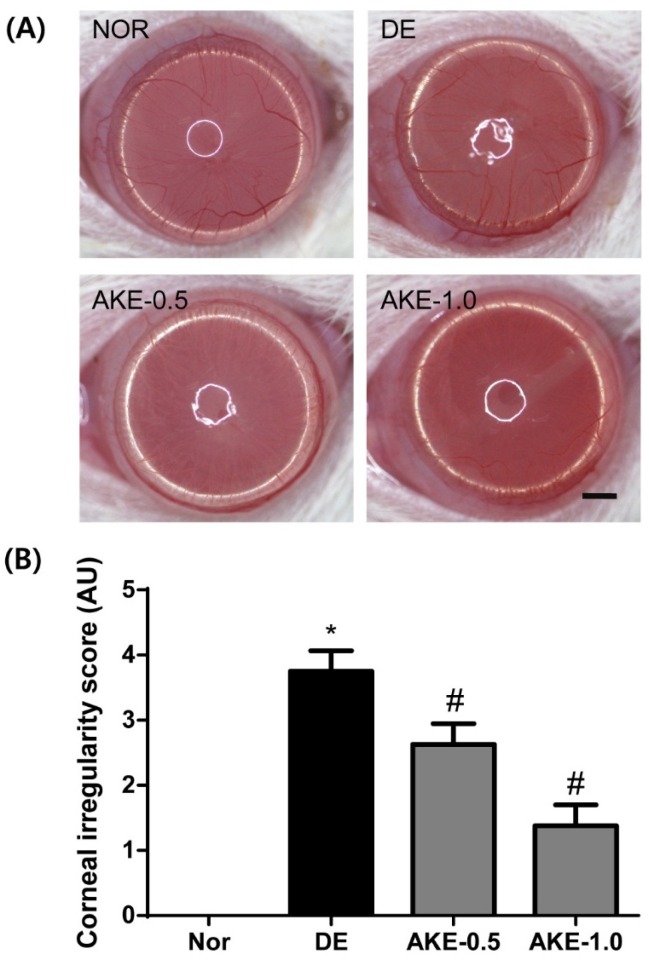
Effects of AKE on corneal irregularity in dry eye mice. (**A**) Reflected images of a white ring from the fiber-optic ring illuminator of a stereomicroscope. Scale bar is 1 mm; (**B**) Corneal irregularity was graded according to the number of distorted quarters in the reflected white ring as follows: 0, no distortion; 1, distortion in one quarter; 2, distortion in two quarters; 3, distortion in three quarters; 4, distortion in all four quarters; 5, severe distortion, in which no ring could be recognized. The values in the bar graph represent the mean ± SE, *n* = 10. * *p* < 0.05 vs. normal mice, # *p* < 0.05 vs. vehicle-treated dry eye mice.

Dry eye disease often results in alterations of corneal epithelial barrier function and tear film integrity. Because the blink response of the mouse is infrequent, it is technically difficult to measure their tear break-up time. Therefore, the distortion of reflected ring-shaped light on the corneal surface can be regarded as the alteration of tear film homeostasis [15]. Mice in the DE group showed increased corneal irregularity (Figure 4A,B). However, AKE dose-dependently improved the corneal smoothness score at five days after application, suggesting that AKE is useful for maintaining tear film integrity.

### 3.5. AKE Inhibits Corneal Mucin Alteration

**Figure 5:**
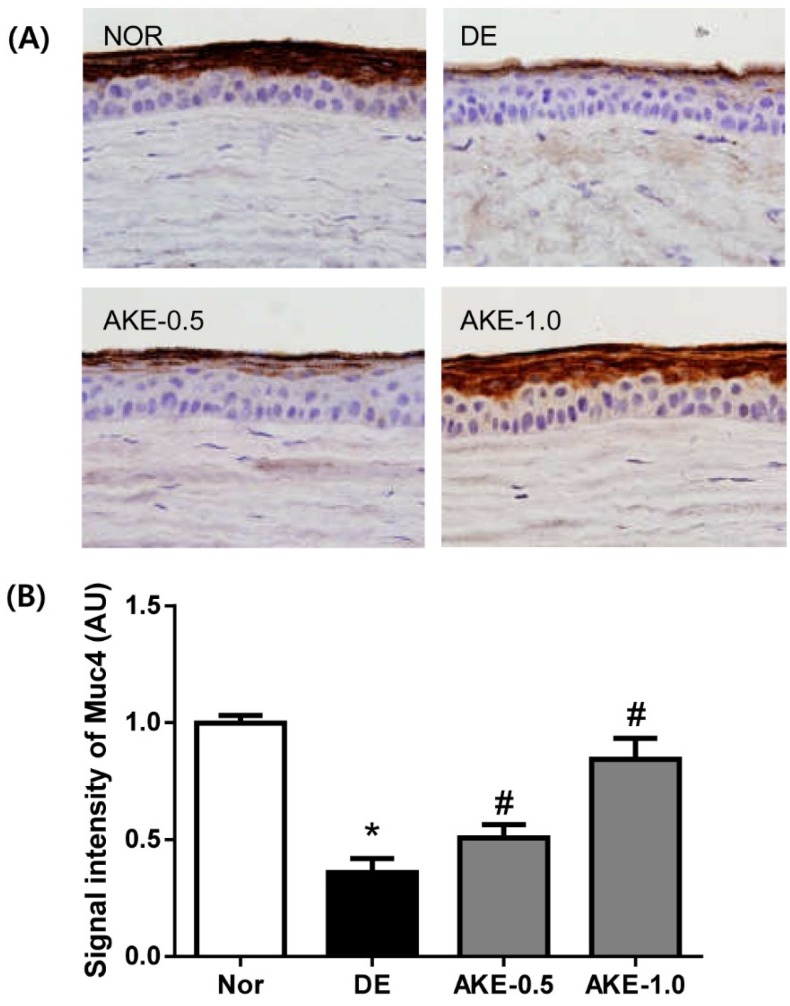
Effects of AKE on the expression level of Muc4 on the ocular surface. (**A**) Immunohistochemical staining for Muc4; (**B**) Morphometric analysis of the Muc4-positive signal density in corneal sections from each group. The values in the bar graph represent the mean ± SE, *n* = 10. * *p* < 0.05 vs. normal mice, # *p* < 0.05 vs. vehicle-treated dry eye mice.

To investigate whether topical application of AKE on the eye induces mucin secretion, we performed immunostaining for Muc4 in corneal sections. As shown in Figure 5, the expression level of Muc4 in vehicle-treated DE mice was significantly reduced compared with that in normal control mice. However, a dose-dependent increase in the expression levels of Muc4 was observed in AKE-treated mice. These findings clearly indicate that AKE accelerates the secretion of Muc4 on the ocular surface.

### 3.6. AKE Suppresses the Expression of TNF-α

**Figure 6:**
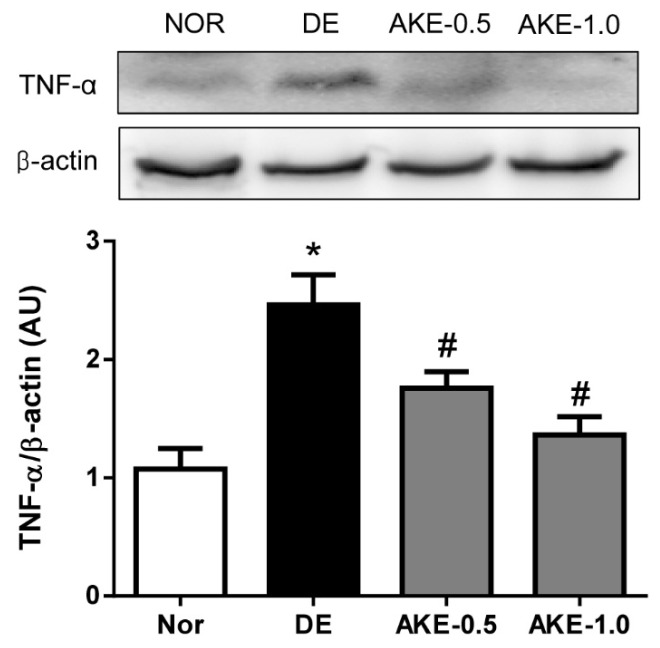
Effects of AKE on the expression level of TNF-α in corneal and conjunctival tissues. The expression of TNF-α was assessed by Western blot analysis. Quantification of relative protein levels, compared with β-actin (a loading control). The values in the bar graph represent the mean ± SE, *n* = 5. * *p* < 0.05 vs. normal mice, # *p* < 0.05 vs. vehicle-treated dry eye mice.

The expression level of the inflammatory cytokine TNF-α was evaluated by immunoblot analysis. As shown in Figure 6, the level of TNF-α in the corneal and conjunctival tissues of mice in the DE group significantly increased compared with that in normal control mice. However, the expression level of TNF-α was significantly reduced in mice treated with AKE. These findings revealed that AKE decreased the inflammatory reaction in the mice with dry eye.

## 4. Discussion

Dry eye disease is a common and multifactorial disease of the ocular surface. It has been widely recognized that the characteristics of pathological alteration in dry eye include inflammation of the ocular surface, corneal epithelial breakage and conjunctival goblet cell loss. Numerous animal models of dry eye syndrome have been developed to investigate the pathophysiology of dry eye and the efficacy of drug candidates. Recently, lacrimal gland excision has been commonly used as a reproducible experimental model that results in dry eye symptoms, including reduced tear volume, increased corneal surface irregularity, disruption of corneal epithelial barrier function and decreased conjunctival goblet cell density [16,17,18]. In the present study, to investigate the therapeutic effect of AKE on dry eye, we clearly confirmed the generation of experimental dry eye three days after excision of the exorbital lacrimal gland and then initiated treatment with AKE from day 3 to day 7 after excision. The topical application of AKE improved the clinical symptoms of dry eye, such as the reduction of tear secretion and corneal barrier disruption, in the exorbital lacrimal gland–excised mice. In addition, the topical application of AKE promoted the secretion of mucin on the ocular surface. These results indicated for the first time that the topical application of AKE improves the clinical outcomes of dry eye syndrome.

Aqueous tears are important for maintaining tear film homeostasis. Only a few drugs, such as diquafosol sodium, can be used to facilitate the production of tear fluid in patients with dry eye [6]. Therefore, we are trying to develop novel agents as therapeutic options for dry eye. Our data clearly showed that AKE increased tear secretion in exorbital lacrimal gland–excised mice in a dose-dependent manner. These results indicate the beneficial effect of AKE on the ocular surface and suggest that AKE might be a therapeutic option.

Currently, the precise pathophysiology of dry eye syndrome is not entirely understood. Recently, it has been recognized that ocular mucin plays a prominent role in the progression of dry eye. Mucins are transmembrane glycoproteins that are detected at the apical surface of all mucosal epithelial cells. They can be further classified as (a) secreted gel-forming mucins and (b) membrane-bound mucins [19]. Membrane-bound mucins have a single transmembrane domain including a large glycosylated extracellular domain, a helical transmembrane domain and a short cytoplasmic tail, and these mucins are expressed in the apical cell membranes of the epithelium [20]. Ten types of membrane-bound mucins have been revealed, and each mucin has a different biological activity. Muc1, Muc4 and Muc16 are detected in the ocular surface epithelial cells [21,22]. On the ocular surface, mucins have multiple functions, such as protection, barrier function and lubrication of the ocular epithelium. It is well known that dry eye is associated with alterations in the expression of mucin proteins and genes [23,24]. Our study clearly showed that AKE increases the expression level of cell surface–associated Muc4 on the ocular surface, suggesting that AKE might help to maintain the apical surface of the corneal epithelium.

In human subjects, optical coherence tomography demonstrated that the thickness map of the corneal epithelium of dry eye was thinner than that of normal eyes [25]. In several previous reports, dry eye disease decreased corneal epithelium thickness in animal models [26,27]. In our present study, we observed thinning of the corneal epithelium of dry eye mice in the immunohistochemical staining for Muc4. Lacrimal gland excision–induced dry eye might lead to the reduction of corneal epithelium thickness in dry eye mice, which is consistent with the findings of Hirayama et al. [28]. However, AKE ameliorated the thinning of the corneal epithelium induced by lacrimal gland excision.

Based on these results, the presumed mechanism underlying the effects of AKE in the treatment of dry eye includes maintenance of tear film integrity by promoting tear fluid and mucin secretion. We performed standardization of AKE and identified amygdalin as a major compound. Although there are no reports on the effects of apricot kernel or amygdalin on ocular diseases, Nabavizadeh et al. reported that amygdalin could help to maintain gastric mucosal integrity in an experimental gastric ulcer model [29]. Nitric oxide is an important mediator of mucin secretion from goblet cells [30]. Amygdalin can induce the generation of gastric mucosal nitric oxide [29]. These findings suggest that the effect of AKE may be ascribed to the effects of its bioactive compound, amygdalin. However, it is difficult to identify which ingredient in AKE is the bioactive compound that acts on dry eye. In the future, studies should be conducted to determine the bioactive compounds of AKE that are useful for treating dry eye diseases.

## 5. Conclusions

In summary, AKE can help to maintain tear film integrity under dry eye conditions. AKE clearly improved dry eye symptoms by promoting the secretion of tear fluid and mucin. Therefore, this study indicates that AKE may serve as a beneficial agent for treating dry eye disease.

## Figures and Tables

**Table 1:** Calibration data for an amygdalin standard.

Compound	Linear Range (μg/mL)	Regression Equation ^a^	Correlation Coefficient (*R*^2^)
Amygdalin	12.5–200	*y* = 2.1113*x* + 7.35	0.9999

^a^ *y*: peak area (mAU) of component; *x*: concentration (μg/mL) of component.

**Table 2:** Content of amygdalin in the apricot kernel extract.

Sample	Amygdalin Content (mg/g) ^a^
Apricot kernel extract	127.34 ± 0.99

^a^ The value is expressed as mean ± standard deviation(SD) (*n* = 3).
